# Feasibility of Known RNA Polymerase Inhibitors as Anti-SARS-CoV-2 Drugs

**DOI:** 10.3390/pathogens9050320

**Published:** 2020-04-26

**Authors:** Ujjwal Neogi, Kyle J. Hill, Anoop T Ambikan, Xiao Heng, Thomas P. Quinn, Siddappa N. Byrareddy, Anders Sönnerborg, Stefan G. Sarafianos, Kamal Singh

**Affiliations:** 1 Division of Clinical Microbiology, Department of Laboratory Medicine, Karolinska Institute, 14186 Stockholm, Sweden; ujjwal.neogi@ki.se (U.N.); anoop.ambikan@ki.se (A.TA.); 2 Department of Molecular Microbiology and Immunology, University of Missouri, Columbia, MO 65211, USA; kjhy8c@mail.missouri.edu (K.J.H.); Anders.sonnerborg@ki.se (A.S.); 3 Bond Life Sciences Center, University of Missouri, Columbia, MO 65211, USA; 4 Department of Biochemistry, University of Missouri, Columbia, MO 65211, USA; hengx@missouri.edu (X.H.); QuinnT@missouri.edu (T.P.Q.); 5 Department of Pharmacology and Experimental Neuroscience, University of Nebraska Medical Centre, Omaha, NE 68198, USA; sid.byrareddy@unmc.edu; 6 Division of Infectious Diseases, Department of Medicine Huddinge, Karolinska Institute, 14186 Stockholm, Sweden; 7 Laboratory of Biochemical Pharmacology, Department of Pediatrics, Emory University School of Medicine, Atlanta, GA 30322, USA; stefanos.sarafianos@emory.edu; 8 Shift Pharmaceuticals, Overland Park, KS 66211, USA

**Keywords:** coronavirus, SARS-CoV, MERS-CoV, SARS-CoV-2, COVID-19, RNA polymerase, nsp12

## Abstract

Coronaviruses (CoVs) are positive-stranded RNA viruses that infect humans and animals. Infection by CoVs such as HCoV-229E, -NL63, -OC43 and -HKU1 leads to the common cold, short lasting rhinitis, cough, sore throat and fever. However, CoVs such as Severe Acute Respiratory Syndrome Coronavirus (SARS-CoV), Middle East Respiratory Syndrome Coronavirus (MERS-CoV), and the newest SARS-CoV-2 (the causative agent of COVID-19) lead to severe and deadly diseases with mortality rates ranging between ~1 to 35% depending on factors such as age and pre-existing conditions. Despite continuous global health threats to humans, there are no approved vaccines or drugs targeting human CoVs, and the recent outbreak of COVID-19 emphasizes an urgent need for therapeutic interventions. Using computational and bioinformatics tools, here we present the feasibility of reported broad-spectrum RNA polymerase inhibitors as anti- SARS-CoV-2 drugs targeting its main RNA polymerase, suggesting that investigational and approved nucleoside RNA polymerase inhibitors have potential as anti-SARS-CoV-2 drugs. However, we note that it is also possible for SARS-CoV-2 to evolve and acquire drug resistance mutations against these nucleoside inhibitors.

## 1. Introduction

Human coronaviruses (CoVs) such as HCoV-229E, NL63, OC43 and HKU1 primarily infect the upper respiratory and gastrointestinal tract, causing mostly mild diseases. However, some CoVs such as SARS-CoV and MERS-CoV cause severe respiratory diseases (SARS and MERS) that result in ~10% to ~35% mortality [1,2,3,4,5]. SARS-CoV caused a pandemic in 2003 with 774 deaths worldwide [6,7,8]. MERS-CoV was first reported in June 2012, and according to a World Health Organization (WHO) report, there were 2494 laboratory-confirmed cases of MERS, including 858 associated deaths, mostly from Middle East countries [9]. However, the current outbreak of SARS-CoV-2, the causative agent of Coronavirus Disease 2019 (COVID-19) has claimed thousands of lives since the first reported case in December, 2019, in Wuhan city, China [10,11,12]. To date, there are no approved vaccines or anti-SARS-CoV/MERS-CoV drugs available. Hence, studies on the discovery of small molecule drugs against SARS-CoV, MERS-CoV and other related future pathogenic coronaviruses are of high importance [13].

CoVs belong to the family *Coronaviridae* of the *Nidovirales* order. CoVs have been divided into α, β, γ and δ-coronavirus genera [14]. The β CoVs have been further divided into four lineages (A–D) [15]. Phylogenic analysis shows that both SARS-CoV and SARS-CoV-2 belong to lineage B of β CoVs [16,17], whereas MERS-CoV belongs is lineage C, and the well-studied mouse hepatitis virus (MHV) in lineage A [18,19,20]. An example of lineage D is Rousettus bat coronavirus HKU9 [21]. Coronaviruses are the largest (26.2 to 31.7 kb) positive [or (+)] sense single stranded RNA viruses. The polyadenylated and capped RNA genome [5,22] has multiple open reading frames (ORFs). The 5′-most two-third of the genome contains ORF1a and ORF1b that encode polyproteins pp1a and pp1ab (made through a −1 ribosomal frameshift during translation), which are cleaved to form the non-structural proteins (nsp) [23,24,25,26,27,28,29,30]. The structural proteins are expressed as subgenomic RNAs and individual RNAs (genomic and subgenomic) are translated to yield only the protein encoded by the 5’-most ORF [31]. These polyproteins are processed by coronavirus-encoded papain-like proteinases (PL^pro^; within nsp3) [32] and nsp5 (3CL^pro^) [5,24,25,33,34,35,36] to yield up to 16 nsps with diverse functions [31,37,38,39,40]). The assembled replication-transcription complex (RTC) binds at the 3′ untranslated region and synthesizes a negative sense (-) RNA template complementary to the genomic RNA, as well as subgenomic (-) strand RNAs with common 5′ ends and leader complementary sequences at the 3′ ends. The (-) RNAs are used as templates to synthesize full-length RNA packaged into virions and a nested set of (+) strand subgenomic mRNAs [31,37,38,39,40].

Nearly 932 amino acids long, nsp12 (RNA polymerase) of CoVs is an essential component of the RTC [26]. nsp12 is a product of pp1ab polyprotein, and serves as the main RNA-dependent RNA polymerase (RdRp) [24,41]. Approximately 500 C-terminal amino acids of nsp12 constitute the RNA polymerase domain. The N-terminal extension (~400 amino acids) of nsp12 is unique to *Nidovirales*. This domain has been proposed to have a nucleotidyltransferase activity, and therefore has been termed as the nidovirus RdRp-associated nucleotidyltransferase (NiRAN) domain [42]. A cryoEM structure of SARS-CoV nsp12 in complex with nsp7 and nsp8 was reported in 2019 [43]. Very recently, a preprint describing the structure of SARS-CoV-2 nsp12 has appeared [44]. Apparently, this structure has strong similarity to that of SARS-CoV nsp12 (root-mean-square deviation (RMSD) of 0.82 Å for 1078 Cα atoms, including nsp7 and nsp8 Cα atoms) [44]. These two cryoEM structures reveal that the overall architecture of the nsp12 polymerase domain resembles canonical RNA polymerases, and assumes a right-hand conformation with structural units reminiscent to fingers, palm and thumb [43]. 

Historically, viral polymerases have proven to be attractive targets for antiviral therapy. HIV-1 reverse transcriptase (HIV-1 RT), hepatitis B virus (HBV) polymerase (HBV pol), and hepatitis C virus (HCV) replicase (NS5B) are some of the notable examples of antiviral targets. All currently recommended first-line antiviral therapies consist of nucleoside analogs that target these viral polymerases. Additionally, nucleoside analogs such as ribavirin and 5-fluorouracil (5-FU) have been approved as broad-spectrum antiviral drugs. Whereas ribavirin in-combination with interferon-α2b, has shown potential for treatment of MERS-CoV infection in rhesus macaques [45], while resistance mutations under 5-FU pressure have been determined in MHV nsp12 [46]. Additionally, gemcitabine, a nucleoside analog and a well-known cancer drug, has been identified as inhibiting SARS and MERS-CoVs [47]. 

The emergence of recent pandemic COVID-19 has compelled researchers and clinicians to explore novel broad-spectrum drugs as inhibitors of SARS-CoV-2. Thus, nucleoside analogs favipiravir (T-705) [48,49] and remdesivir [49,50,51,52] have shown inhibitory potential against SARS-CoV-2. Owing to the potential of remdesivir, the United States Food and Drug Administration (FDA) has granted an orphan drug status on 23 March 2020 so that it can be used in clinics, and a clinical trial has started at the University of Nebraska Medical Center, Omaha, NE (NCT04280705). In order to explore the feasibility of broad-spectrum nucleoside inhibitors of RNA polymerases as potential inhibitors of SARS-CoV-2, we used comparative molecular modeling, docking and bioinformatics to assess these compounds as potential inhibitors of nsp12. More specifically, we present the feasibility of remdesivir, 5-FU, ribavirin, and favipiravir (T-705) as anti-SARS-CoV-2 compounds. 

## 2. Results and Discussion

### 2.1. Sequence Conservation Among SARS-CoV, MERS-CoV and SARS-CoV-2 nsp12 Proteins

Nucleoside analog inhibitors are administered as compounds containing a nucleic acid base with modified sugar moiety. These compounds are metabolized into their triphosphate (TP) form by cellular kinases, becoming the *bona fide* substrates of nucleic acid polymerases. The nucleic acid polymerases contain conserved motifs that participate in nucleoside-TP (NTP) binding [53]. First, we assessed sequence conservation in the NTP-binding motifs using available nsp12 sequences of SARS-CoV, MERS-CoV and SARS-CoV-2.

**Figure 1:**
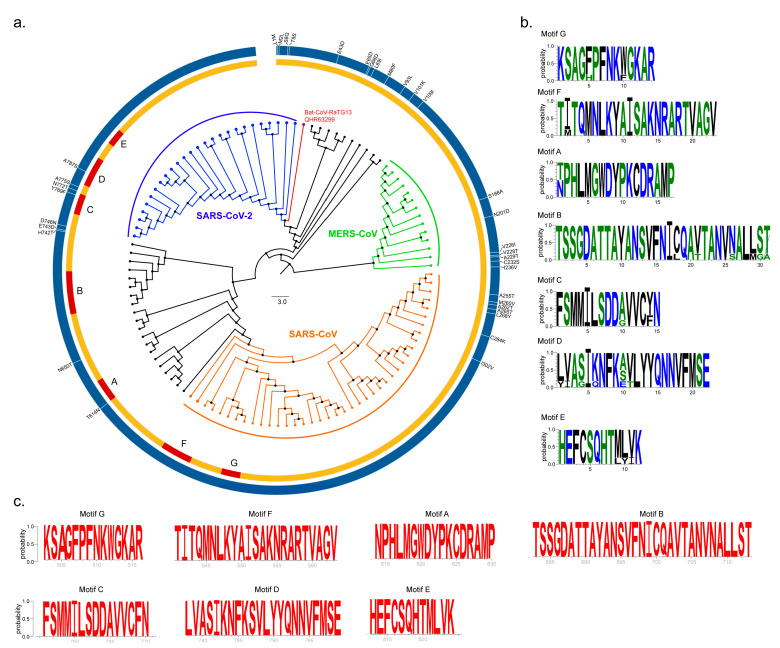
Phylogenetic analyses and sequence conservation. (**a**) Phylogenetic analysis was performed by the MEGA X software using the nsp12 sequences of Bat CoV (Black), Severe Acute Respiratory Syndrome Coronavirus (SARS-CoV) (Orange), Middle East Respiratory Syndrome Coronavirus (MERS-CoV) (green) and SARS-CoV-2 (blue). The Bat CoV-RaTG13 that was proposed to be the origin of the SARS-CoV-2 is marked in red. The Circos plot was created using Circos software package (v0.69-8). The amino acid changes between consensus SARS-CoV-2 compared to consensus SARS-CoV were identified by multiple sequence alignment and denoted as vertical bars on the Circos plot. (**b**) The seven conserved RNA-dependent RNA polymerase (RdRp) motifs (A–G) are as denoted by the sequence logo (WebLogo v3). (**c**) Sequence conservation of SARS-CoV-2 nsp12 motifs are shown as sequence logos (red).

We then conducted a comprehensive phylogenetic analysis of nsp12 proteins using available sequences from SARS-CoV (n = 40), MERS-CoV (n = 14) and SARS-CoV-2 (n = 26) along with Bat CoV (n = 31) (Figure 1a). Our analyses showed that SARS-CoV-2 is closely related to the Bat CoV-RaTG13 strain, which is consistent with earlier reports [8]. The majority of sequence variation was present in the N-terminal region of nsp12, belonging to NiRAN and Interface domains (the description of the Interface domain in presented in the following section). The polymerase domain (amino acid residues 399–932) is highly conserved among all SARS-CoV-2 nsp12 proteins with only nine substitutions (T614N, N650S, H742T, E743D, D746N, Y769F, N772T, A775S, A787S) with respect to SARS-CoV (Figure 1a). The RdRp motifs (A to G) are highly conserved in the SARS-CoV, MERS-CoV and SARS-CoV-2 strains (Figure 1b). SARS-CoV-2 RdRp motifs are fully conserved within currently available strain sequences (n = 179) (Figure 1c). This is further supported with the large number of sequences (n = 4551 as of 20 April 2020) available in the Genomic epidemiology of hCoV-19 (https://www.gisaid.org/epiflu-applications/next-hcov-19-app/).

### 2.2. Structure of SARS-CoV-2 nsp12 

Based on the cryoEM structure, nsp12 has been divided into three structural regions: (i) the NiRAN domain (residues 117–250), (ii) Interface domain (residues 251–398), and (iii) polymerase domain (residues 399–919) [43]. The recently reported structure of SARS-CoV-2 nsp12 showed the presence of a newly identified N-terminal β-hairpin, which interacts with the palm subdomain of nsp12. We modeled SARS-CoV-2 nsp12 structure using the available cryoEM structure of SARS-CoV nsp12 (PDB file 6NUR [43]). The protein sequence of SARS-CoV nsp12 has 99.9% identity with GZ02 isolate (GenBank accession number AAS00002). The SARS-CoV-2 nsp12 sequence that we used in homology modeling was taken from isolate WIV05 (GenBank accession number QHR63269). The two sequences (nsp12 from SARS-CoV and SARS-CoV-2) have ~94% identity with the most sequence variation existing in the N-terminal β-hairpin, NiRAN, and Interface domains (Figure 1a). 

**Figure 2:**
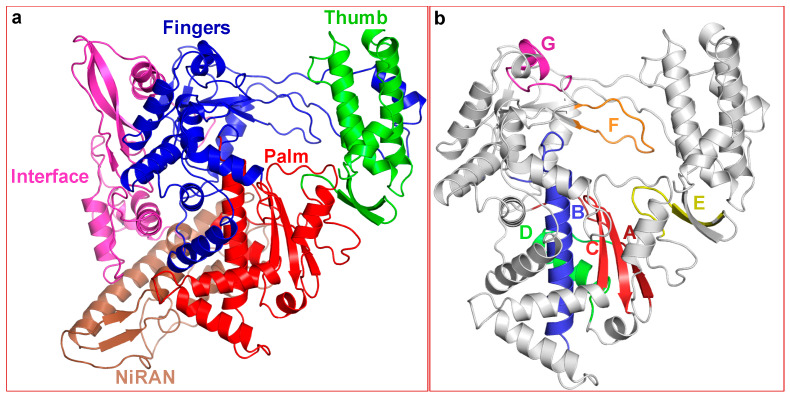
Homology-derived molecular model of SARS-CoV-2 nsp12 and the location of conserved RNA polymerase motifs. (**a**) Overall folding of SARS-CoV-2 nsp12. The fingers, palm and thumb are colored in blue, red and green, respectively. The NiRAN and Interface domains are colored brown and magenta, respectively. (**b**) The polymerase domain of nsp12. Location of the conserved motifs in SARS-CoV-2 conserved motifs. Motifs A and C, which harbor the catalytic site residues are colored dark-red and red, respectively. Motifs B, D, E, F and G are colored as blue, green, yellow, orange and magenta, respectively.

The modeled structure of SARS-CoV-2 nsp12 (Figure 2) superposed extremely well on to the cryoEM structure of SARS-CoV nsp12 (RMSD of <0.5 Å for 802 Cα atoms). Nine non-conserved residues in the polymerase domain are located at the surface of nsp12 distal to the polymerase active site (D621, D760 and D761). All conserved RdRp motifs (A–G) [13] were easily identifiable in the modeled structure of SARS-CoV-2 nsp12 (Figure 2b). One of the unusual features of modeled SARS-CoV-2 nsp12 is the partial β-strand structure of motif A that contains one carboxylate (D621) of the catalytic triad. In fact, a similar conformation is present in the cryoEM structure of SARS-CoV nsp12 [43]. Motifs A and C are known to form a three-stranded β-sheet composed of one strand from motif A and two strands from motif C in both RdRps and DNA-dependent DNA polymerases. However, the crystal structures of poliovirus RdRp (3Dpol) and enterovirus 71 RdRp (3Dpol) elongation complexes showed subtle conformational changes in the palm subdomain (called the ‘active site closure’) and that the presence of incoming substrate induces inter-β-strand hydrogen bonds required for classification as β-strand (reviewed by Peersen [54]). Therefore, the partial β-strand structure of motif A is expected to adopt a complete β-strand conformation in the complex consisting of primer-template (pt) and the nucleoside triphosphate (NTP).

### 2.3. Nucleoside RNA Polymerase Inhibitors

#### 2.3.1. Remdesivir 

**Figure 3:**
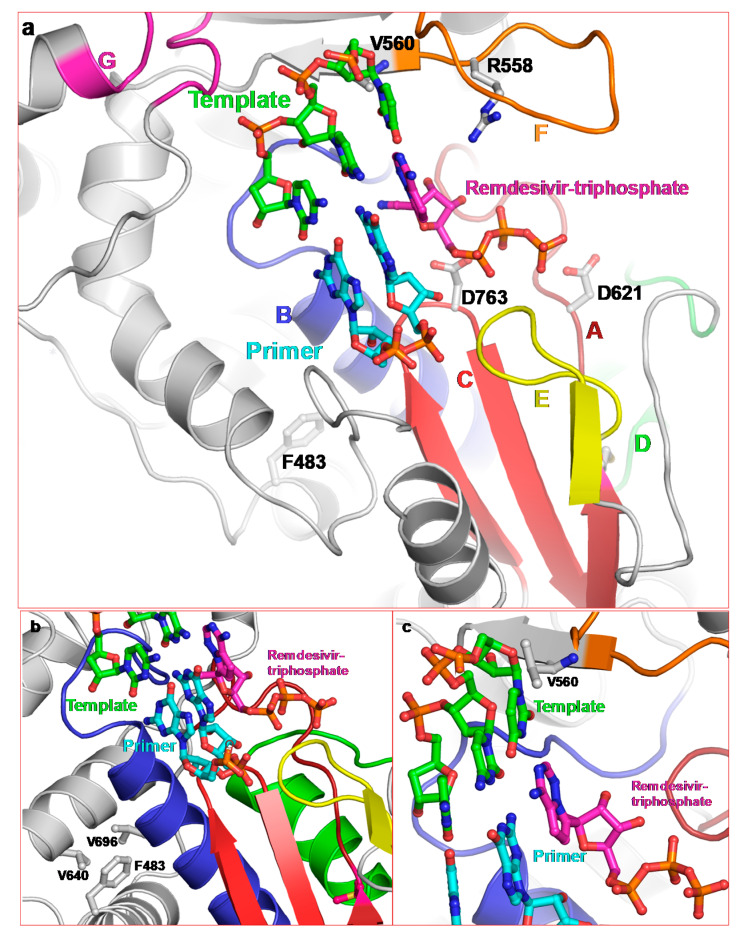
Proposed binding of remdesivir-triphosphate and location of remdesivir resistance residues. (**a**) Molecular model of nsp12/pt/remdesivir-TP showing the proximity of conserved motifs to the substrate. (**b**) F483 forms hydrophobic interactions with motif B residues V696 and V640, and (**c**) position of V560 close to the templating nucleotide.

Remdesivir (GS-441524) is a 1′-cyano 4-aza-7,9-dideazaadenosine C-adenosine nucleoside analog. It is a broad-spectrum RNA polymerase inhibitor that has been shown to inhibit human and mouse CoVs [52]. More importantly, remdesivir has been shown to inhibit SARS-CoV-2 in vitro [48,55]. A recent report showed that compassionate-use of remdesivir showed improvement in 68% of COVID-19 patients [56]. The antiviral activity of remdesivir against SARS-CoV-2 is not surprising as it is a nucleoside analog and expected to bind at the NTP-binding site, which is highly conserved among SARS-CoV, MERS-CoV and SARS-CoV-2 nsp12 polymerases (Figure 1b). Except motifs D and G, all other motifs either directly participate in NTP binding/hydrolysis or are spatially located in close vicinity of the remdesivir-TP binding site. A molecular model consisting of enzyme, remdesivir-TP and RNA (pt) is shown in Figure 3a.

Motifs A and C harbor catalytic site carboxylates and motif B binds the base/sugar moiety of the NTP. Both are close to remdesivir-TP (Figure 3a). Motif E, which is in the vicinity of the NTP binding pocket, is present only in RNA polymerases, and has been termed as ‘primer grip’ [6,7]. This motif is also in close proximity to remdesivir-TP. Motif F contains a highly conserved basic residue, which interacts with the TP moiety of NTP. We also identified R558 in the SARS-CoV-2 nsp12/pt/remdesivir-TP model as the conserved motif F basic residue, which interacts with the β-phosphate (Figure 3a). 

Resistance to remdesivir has been demonstrated in in vitro passage assays [57]. Two mutations (F476L and V553L) in MHV nsp12 appeared after 23 passages. Amino acid residues F476 and V553 counterparts are numbered F483 and V560, respectively in the cryoEM structure of SARS-CoV. Hence, hereafter we will refer to these residues according to their numbering in the cryoEM structure of SARS-CoV nsp12 (i.e., F483 and V560). Both F483 and V560 are absolutely conserved in α-, β-, and γ-CoVs, and belong to the fingers subdomain of nsp12. Their locations relative to remdesivir-TP are shown in Figure 3b,c, respectively. V560 is proximal to motif F. In our model of nsp12/pt/remdesivir-TP complex, V560 is close to the template nucleotide that is base-paired with remdesivir-TP (or incoming NTP). Topologically equivalent valine (V181) interacts with the templating base in the crystal structures of foot-and-mouth disease virus (FMDV) 3Dpol consisting of E/pt/ATP [58] and Coxsackievirus 3B 3Dpol [54]. Therefore, mutation V560L in nsp12 may alter the position of the template nucleotide and reduce the binding of remdesivir-TP, thereby imparting resistance to remdesivir. 

F483 is located adjacent to motif B and forms hydrophobic interactions with V640 and V696 of motif B. Mutation to L483 results in a shorter side chain yet maintains hydrophobic interactions with neighboring V640 and V696. It is possible that subtle changes in the hydrophobic interactions may assist in the known mechanism of active site closure in RdRps [29,30] to enhance fidelity of nsp12, i.e., preferential selection of NTP over remdesivir-TP. 

#### 2.3.2. 5-fluorouracil (5-FU)

The polymerase domain of SARS-CoV nsp12 has a high structural homology with picornavirus 3Dpol [43]. Hence, we reasoned that the nucleoside analogs, known to inhibit the well-studied FMDV 3Dpol, might inhibit nsp12. The mechanism of inhibition of two major nucleoside analogs, 5-FU and ribavirin, has been structurally well studied [58,59,60]. Below, we discuss the feasibility of 5-FU and ribavirin for the inhibition SARS-CoV-2 nsp12. 

5-FU is a pyrimidine analog that has been used in clinics as an anti-cancer drug for many years [61,62]. Additionally, it is a mutagen for several viruses [46,63]. Incorporation of 5-FU-monophosphate (5-FUMP) into the viral genome by RdRps leads to error catastrophe [64,65]. Efficient extinction of FMDV has been achieved by 5-FU in combination with guanidine hydrochloride and heparin [66]. Additionally, 5-FU after its conversion to 5-FU-triphosphate (5-FUTP) blocks initiation of FMDV RNA synthesis and therefore functions as an initiation inhibitor [67]. Mutations in RdRp enzymes under 5-FU pressure impart fitness loss in the absence of 5-FU, but confer a fitness gain in presence of 5-FU. Most RNA viruses do not possess a proofreading activity. Therefore, these viruses overcome the effect of mutagens by selecting resistance mutations that enhance the fidelity RNA synthesis [68]. 

CoVs also encode nsp14, which acts as a proofreading enzyme. Deletion of nsp14 renders SARS-CoV sensitive to 5-FU [69]. Furthermore, mapping the mutations affecting fidelity in Coxsackievirus B3 onto the MHV nsp12 molecular model, and introducing these mutations into MHV with [nsp14-ExoN(+)] or without [nsp14-ExoN(−)] exonuclease activity, two mutations (V560I and M618F) were identified that conferred resistance to 5-FU [46]. Mutation at nsp12 codon 560 (V560L) has also been reported for remdesivir (discussed above). Therefore, resistance to 5-FU by mutation at V560 may occur through the repositioning of templating nucleotide, which, in turn may alter the selectivity of the enzyme for UTP over 5-FUTP. 

We have previously reported that mutation V173I in FMDV 3Dpol enhances selectivity of UTP over 5-FUTP [70]. Using pre-steady state kinetics, we showed that V173I mutation in FMDV 3Dpol enhances the selectivity of UTP over 5-FUTP by ~3.2-fold compared to the wild-type enzyme. The selectivity of UTP over 5-FUTP by V173I 3Dpol was primarily due to the increase in the dissociation of 5-FUTP from the elongation complex, which resulted in restricted 5-FUMP incorporation [70]. FMDV containing V173I survived the mutagenic activity of 5-FU by compensating for the increase in A→G and U→C transitions that the wild-type virus endures in the presence of 5-FU [70]. Compensation in the mutant virus entails an increase of G→A and C→U transitions in the presence of 5-FU, which approximates the mutational pattern to that of the wild-type virus replicating in the absence of 5-FU [70]. Due to the fact that CoVs contain an exonuclease enzyme, the change in NTP selectivity may be a primary mechanism of 5-FU resistance, since the misincorporation of 5-FUMP would most likely be corrected by the nsp14 exonuclease. 

**Figure 4:**
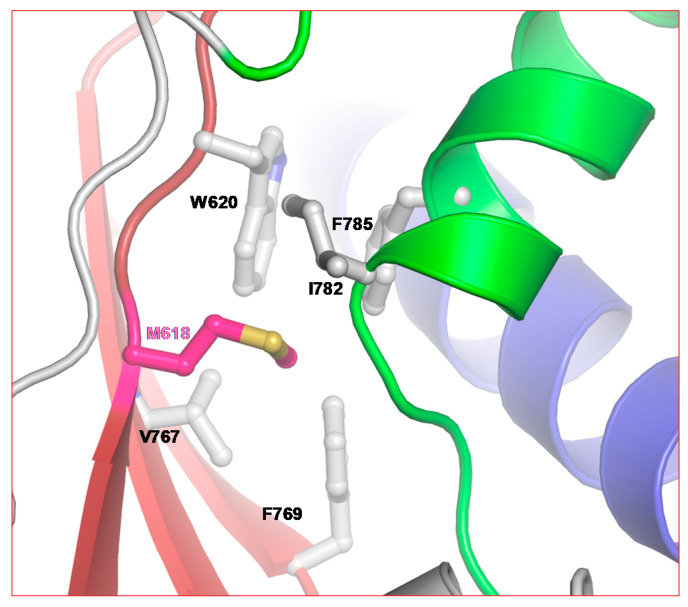
Position of 5-FU resistance mutation in CoV. M618 of SARS-CoV-2 is part of the highly conserved motif A. Note the hydrophobic cluster of residues around M618.

5-FU resistance mutation position M618 belongs to the conserved motif A. As described above, the active site closure mechanism of RdRps serves as a fidelity control [71]. A comparison of RdRp palm domains suggests that all (+) strand RNA viruses use this active site closure mechanism to optimize the fidelity of RNA synthesis [14]. As shown in Figure 4, M618 is clustered among hydrophobic residues emanating from motifs A (dark-red), C (red), and D (green). Mutation M618F will result in the introduction of a bulky side chain (phenylalanine), which is also more hydrophobic than methionine. This may lead to a subtle change in the palm-based closure mechanism of RdRps (in the case of nsp12) and therefore enhance the fidelity of RNA synthesis. M618 is topologically equivalent to position I230 in Coxsackievirus and F230 of poliovirus. Mutation at this position has been shown to affect the fidelity of the RdRp [14]. Hence, it is possible that selection of M618F in the presence of 5-FU is related to the fidelity of nsp12.

#### 2.3.3. Ribavirin

Ribavirin (1-β-d-ribofuranosyl-1,2,4-triazole-3-carboxamide) is an FDA approved antiviral drug. It is one of the most widely used broad-spectrum inhibitors of RNA viruses. Similar to 5-FU, it is mutagenic for many RNA viruses [64,72,73,74,75,76,77]. Ribavirin has been in clinics for many years to treat HCV (in combination with pegylated interferon) [78], FMDV [79] and poliovirus [80]. Additionally, ribavirin in combination with interferon-α2a or α2b has been shown to inhibit MERS-CoV infection [81,82,83]. Ribavirin triphosphate (RTP) binds at the NTP binding site of FMDV 3Dpol [58]. Due to the fact that NTP binding motifs are highly conserved among RdRps (Figure 1 and Figure 2), RTP is predicted to bind and exert its inhibitory effect on all CoVs, including SARS-CoV-2.

Resistance to ribavirin in different RNA viruses is achieved by mutations in the RdRp coding gene. In poliovirus, FMDV and enterovirus 71, mutations in the 3Dpol (RdRp) confer resistance to ribavirin [84,85,86,87]. HCV develops resistance to ribavirin (when used in combination with pegylated interferon) by blocking downstream signaling actions of STAT1, STAT2, IRF9 and JAK-STAT pathways [88,89], and by mutation in the RdRp gene [90,91]. Mutations G64S and L420A in poliovirus 3Dpol, M296I in FMDV 3Dpol, and F415Y in HCV NS5B have been reported to impart resistance to ribavirin. Mutations at G64 and L123 in enterovirus 71 3Dpol have also been reported to confer ribavirin resistance [87]. 

**Figure 5:**
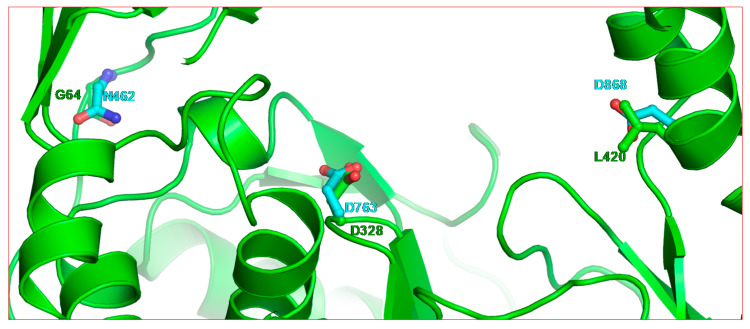
Residue positions of two ribavirin resistance mutations (G64 and L420) in poliovirus 3Dpol relative to the active site residue (D328). The backbone of poliovirus 3Dpol is rendered in green ribbon (PDB entry 3OL6 [92]). The equivalent positions in SARS-CoV-2 nsp12 are shown as cyan residues.

Structurally, ribavirin resistance mutation sites in RNA polymerases do not appear to be in absolutely equivalent positions. A structural alignment showed that G64 in poliovirus 3Dpol is ~17 Å away from the active site, whereas M296 in FMDV 3Dpol is part of the NTP binding site (i.e., within 12 Å). While Y415 in HCV NS5B and L420 in poliovirus 3Dpol are at topologically equivalent positions, they are ~22 Å away in the thumb subdomain. In poliovirus 3Dpol, resistance to ribavirin is achieved by mutations at G64 and at L420 (Figure 5). These two mutation sites are almost posterior to the active site. Residue D868 of nsp12 is topologically equivalent to L420 of poliovirus 3Dpol, whereas N462 (nsp12) can be tentatively assigned as the equivalent to G64 (3Dpol). Both Y415 (of NS5B) and L420 (of poliovirus 3Dpol) interact with the RNA primer strand near the active site [92,93]. Mutation G64S in poliovirus 3Dpol and M296I in FMDV 3Dpol change the fidelity of the two enzymes [94,95], whereas mutation L420A facilitates RNA recombination [86]. These examples suggest that resistance to ribavirin can be achieved by more than one mechanism. It is possible that resistance to ribavirin in SARS-CoV, MERS-CoV and SARS-CoV-2 can develop through mutation at D868 or through yet another unknown mutation and/or mechanism.

#### 2.3.4. Favipiravir (T-705)

Favipiravir (T-705; 6-fluoro-3-hydroxy-2-pyrazinecarboxamide) is a broad-spectrum inhibitor of RNA viruses [96,97], including Ebola virus [98], Crimean-Congo Hemorrhagic Fever [99], Lassa virus [100] and Chikungunya virus [101]. It was also approved as an anti-influenza drug in 2014 in Japan and, more recently, the Italy AIFA and China FDA have approved its use for treatment of COVID-19. 

Favipiravir was discovered by chemical modification of a pyrazine analog (T-1106) [96]. After entering the cell, favipiravir is metabolized into the triphosphate form (T-705-RTP) that is recognized by RdRps. T-705-RTP competes with ATP and GTP [101], suggesting that it is recognized as a purine analogue. In contrast to many nucleoside inhibitors, favipiravir does not have a sugar moiety when administered. Human hypoxanthine guanine phosphoribosyltransferase converts T-705 into its ribose-5’-monophosphate (RMP) prior to formation of T-705-RTP [102]. Mechanisms of inhibition by T-705 have been demonstrated by the chain termination of nascent RNA [103] and by induction of lethal mutagenesis [104,105]. Currently, favipiravir is being evaluated for COVID-19 treatment and the results are awaited.

Resistance to T-705 by RdRps is achieved by mutation of a conserved lysine of motif F. Thus, in chikungunya RdRp, K291R exerts low-level resistance to T-705 [101], and mutation K159R in Coxsackievirus B3 (CVB3) 3Dpol resulted in a nonviable virus [106]. The replication competence of K159R virus was restored by the A239G mutation. Biochemical results suggested that K159R reduced the processivity of CVB3 3Dpol, and the double mutant (K159R/A239G) had low fidelity [106]. The CVB3 K159 equivalent in nsp12 is K548. Currently, it is not known if the mutation of K548 will have effect on favipiravir. However, considering its conserved position, the resistance to favipiravir by SARS-CoV-2 is quite possible.

## 3. Materials and Methods 

### 3.1. Sequence Retrieval and Phylogenetic Analysis

The nsp12 protein sequences of Bat CoV, SARS-CoV, MERS-CoV and SARS-CoV-2 were retrieved using BLASTp (protein-protein BLAST) algorithm with BLOSUM62 matrix. Multiple sequence analysis was performed in AliView software. The ML tree was inferred using RAxML v8.1.20 [107]. The branch supports were computed out of 100 bootstrapped trees. The tree was visualized in FigTree v1.4.4 (http://tree.bio.ed.ac.uk/). The CIRCOS plot was created using Circos software package (v 0.69-8). Amino acid changes in SARS-CoV-2 against SARS-CoV were obtained by pairwise sequence alignment using AliView v1.26 [108].

### 3.2. Molecular Modeling

Homology-derived molecular model of SARS-CoV-2 nsp12 was generated using ‘Prime’ of Schrödinger Suite (Schrödinger LLC, New York, NY, USA) and the cryoEM structure of SARS-CoV [43] (PDB file 6NUR). To generate ternary complex containing enzyme/pt/NTP or enzyme/pt/nucleoside-TP, the pt and RTP from the crystal structure of FMDV 3Dpol [58] (PDB file 2E9R) were extracted and docked into the modeled structure of nsp12. The templating nucleotide was modified as required for the complementarity of the incoming substrate. All the complexes were energy minimized using the Jaguar program of Schrodinger Suite. 

## 4. Conclusions

In conclusion, here we show that the nucleoside inhibitor binding pocket is largely conserved among diverse RNA-dependent RNA polymerases, and that the broad-spectrum nucleoside inhibitors discussed may have potential in COVID-19 treatment. While it is possible that SARS-CoV-2 may evolve with drug resistance mutations against these nucleoside inhibitors, knowledge of potential escape mutants may aid in the development of more specific SARS-CoV-2 inhibitors with a higher resistance barrier. The emerging genomic sequences and structures of SARS-CoV and SARS-CoV-2 nsp12 also offer increasing insight into the design and identification of novel nucleoside inhibitors or small molecules that are specific to SARS-CoV-2 nsp12, and could be used against the current COVID-19 pandemic or in future CoV outbreaks. The in vitro analysis that is presently on-going in the laboratory will provide a better picture of their comparative in vitro potency and resistance profile.

Additionally, the use of these antivirals has an added benefit, as a significant wealth of knowledge already exists regarding their administration, efficacy, toxicity and side effects in humans, which can speed up clinical trials in COVID-19 patients. Inhibitors targeting nsp12 will block the replication of both (+) and (-) strand of the viral genome, which is essential for the formation of a mature and infectious virus.
